# Shifting participatory approach when ideology meets reality: a grounded theory study based on project leaders’ experiences with peer-led sex education programs for and by persons with intellectual disabilities and/or autism

**DOI:** 10.1186/s12978-025-01975-6

**Published:** 2025-03-06

**Authors:** B. Nelson, M. Emmelin, A. Agardh, L. Löfgren, M. Stafström

**Affiliations:** 1 Division of Social Medicine and Global Health, Lund University, 214 28 Malmö, Sweden; 2 Department of Social Work, Malmö University, 206 05 Malmö, Sweden

**Keywords:** Sex education, Peer education, Intellectual disability, Autism, Project leaders, NGO programs

## Abstract

**Background:**

This study explored peer-led sex education for individuals with intellectual disabilities and/or autism (ID/ASD) from the perspective of project leaders within Swedish non-governmental organizations (NGOs). The purpose of this Grounded Theory study was to develop a conceptual model that characterizes the ways in which peer-led sex education is implemented by Swedish NGOs. This was done by exploring what the concept of peer-led sex education means to NGO project leaders, and how they experience, explain and reason about the application of peer education in their daily operations.

**Methods:**

This study conducted 12 qualitative in-depth interviews with project leaders working with peer-led sex education initiatives. Grounded Theory enabled the construction of a conceptual model.

**Results:**

The study identified the core category, "Shifting participatory approach when ideology meets reality," encapsulating project leaders’ experiences in managing peer-led sex education programs. Three distinct approaches were discerned: (1) The Radical approach, where project leaders prioritize empowerment and norm criticism, striving to create an inclusive and equitable environment for individuals with ID/ASD. This approach resonates with Paulo Freire's pedagogy of the oppressed, emphasizing liberation through education. (2) The Pragmatic approach, which navigates the tension between ideology and pragmatism, recognizing the co-dependency between project leaders and persons with ID/ASD. External pressures from funders and the requirements to achieve tangible project outcomes inform this approach. (3) The Skeptical approach, which exhibits caution, doubting the capabilities and willingness of individuals with ID/ASD to challenge societal norms and work equally with people without ID/ASD.

**Conclusions:**

The findings underscore the complexity of peer-led sex education programs and highlight the need for a balanced approach that addresses both ideological aspirations and practical constraints. Empowerment and norm criticism are central to fostering agency and challenging oppressive systems. However, the pragmatic realities of project management and external pressures necessitate a delicate balance. Understanding these diverse approaches can inform the design of more effective initiatives, ultimately contributing to sexual and reproductive health and rights of individuals with ID/ASD.

## Plain Language Summary

This study is about sex education for and by people with intellectual disabilities and/or autism (ID/ASD). This research focuses on peer education programs managed by Swedish non-governmental organizations (NGOs). Through interviews with the program project leaders we discovered that they use three main approaches (1) A Radical Approach that emphasizes empowering peer educators with disabilities as well as challenging societal norms. The aim is to create an inclusive environment where everyone's voice is heard. Here inspiration is drawn from Paulo Freire's idea of using education to liberate and empower marginalized groups. (2) A Pragmatic Approach which tries to find a balance between ideals and the practical demands of running a program and where the need to meet specific goals and fulfill the requirements of funders is recognized. (3) Finally, a Skeptical Approach which is marked by caution and skepticism. In this approach there is uncertainty about whether participants with ID/ASD can challenge societal norms effectively. Awareness of the limitations when running projects with people with intellectual disability may lead to prioritizing stability over empowerment. In conclusion, running peer-led sex education programs for people with ID/ASD is not straightforward. Balancing empowerment and practicality is challenging. Understanding these different approaches can create more effective programs that empower individuals while addressing real-world constraints. Future research should involve the perspectives of peer educators and program participants for a comprehensive view of these programs’ impact and challenges.

## Background

The United Nations Sustainable Development Goals (SDGs) under the principal “Leave no one behind” encompass 17 objectives promoting equality and equity in various domains [1]. In a 2020 Swedish review of the SDGs, persons with disabilities were identified as neglected and the target indicators related to Sexual and Reproductive Health and Rights (SRHR) for SGG3 (Good Health and Well-Being) as well as SDG5 (Gender Equality) had not been met [1]. In Sweden persons with intellectual disability (ID) and/or autism spectrum disorder (ASD) have been systematically restricted from access to SRHR [2, 3]. Comprehensive sexuality education (CSE) is an accepted and relied on resource to understanding ones sexual and reproductive rights as well as what services are available [4, 5]. The Swedish school system has had mandatory sex education since 1955, and the present form of CSE includes includes health and well-being promotion, and informed decision-making. It covers topics such as individual rights, consent, gender power structures, and honor-related violence. Additionally, students learn critical perspectives on media portrayals of sexuality and relationships, including pornography[6]. In Sweden sex education serves as a mechanism to shape what society determines as acceptable values, as well as sexual-social problems [4]. Sweden has a long history of CSE in schools, including special needs schools, although the quality and content of the education varies [7]. Students report that their sexuality is often ignored. One student from this report stated,”’They [teachers] think that we are too immature and then they don’t want to talk about it’” ([8] p. 20). As a result, the report concludes that students miss out on important information about sex and relationships [8]. Once leaving school there is no mandatory CSE for people with ID/ASD, although the Swedish Act concerning Support and Service for Persons with Certain Functional Impariments (LSS) enacted in 1993 provides various forms of support [9]. These include supported housing, personal assistance and adapted workplaces [9]. Some Swedish municipalities, such as the city of Malmö [10], for example, have policies and guidelines for personnel working with sexuality within publicly funded supported housing and workplaces. However, to the authors’ knowledge, no municipality or regional district systematically trains staff in the implementation of these policies. Despite a long history of CSE, adult persons with ID/ASD report lack of sex education and support [8].

Over the past two decades non-governmental organizations (NGOs) have been actively involved in trying to increase the sexual rights of adult persons with ID/ASD supported by government bodies [11, 12]. Several of these initiatives have been based on peer-education, a teaching format whereby persons with similar status and/or ages deliver information to others in their social circles [13]. A key distinction is that the focal group is included in the planning and delivery of the messages [14, 15]. It has been shown that peers are seen as credible sources of information, are linguistically competent, and capable of understanding the focal groups’ situation[15]. However, the design and implementation of peer education have been contested in part due to lack of theoretical underpinnings and long-term behavioral change [15, 16]. Nevertheless, peer education has also been successfully used in sexual health programs geared towards groups that otherwise are difficult to reach [17].

In 2022 Svensson Choudry & Stattin evaluated the success, obstacles and legacy of NGOs’ work with peer education in SRHR among persons with ID/ASD in Sweden [11]. The authors found that projects which had been initiated by the focal group themselves or where the activities had become part of the organizations’ regular activities had been the most successful. Barriers highlighted in this evaluation were the many challenges that the responsible project leaders faced, in particular heavy workload leading to sick leave [11].

Given the varied outcomes of international peer education studies and the absence of a solid theoretical foundation, it is of interest to understand how NGOs in Sweden use peer-led sex education for and by persons with ID/ASD. As highlighted in the 2022 evaluation by Svensson Choudry & Stattin [11], project leaders play a pivotal role in the successful delivery of these programs. To fully harness the benefits of peer education for people with ID/ASD, there is a need to better understand the roles, approaches, and experiences of project leaders in these initiatives.

The purpose of this Grounded Theory study, was to develop a conceptual model that characterizes the ways in which peer-led sex education is implemented by Swedish NGO’s. This was done by exploring what the concept of peer-led sex education means to NGO project leaders, and how they experience, explain and reason about the application of peer education in their daily operations.

## Method

### Study design

The qualitative research methodology of Grounded Theory, as described by Corbin and Strauss, was the analytical approach used in this study [18]. This methodology aims at generating theory or concepts grounded in systematically collected and analyzed data[18]. Corbin and Strauss’s structured approach to identifying patterns, behaviours, and actions [18] make it particularly suitable for the construction of conceptual models rooted in the experiences and narratives of the study participants.

### Study context

The study was conducted in four large cities in central and southern Sweden among project leaders affiliated with NGOs. Five NGOs were identified as NGOs working with peer education for and by people with ID/ASD and SRHR. Of the 12 participants, one was from Uppsala, one was from Stockholm, two were from Gothenburg, and four were from each of the two organizations in Malmo. Methods such as theater, discussion groups, meeting places or workshops are used to address various aspects of SRHR, including safer sex, contraception, consent, sexual violence, sexual norms, identity and relationships. The projects have different titles for the peer educators including educator, expert, volunteer, and actor. Projects are financed through government funding. Three of the NGOs continue to work with the same or similar projects, and two of the NGOs do not currently have projects in the field. All the projects focused on adult persons with intellectual disabilities as the focal population. In the period post data collection and analysis one NGO has expanded to two other cities. Two municipal governmental departments were also using a variation of peer education but as the methods and materials they use were not developed in conjunction with people with ID/ASD and the funding of the work was significantly different, it was determined that the research question would be best answered by only including NGOs. Two NGOs that do not currently have projects in the field were included because both had recently finished their projects and the project leaders were also still working with similar projects. Therefore, these project leaders were available and able to speak to researchers about the peer education SRHR projects.

### Sampling of participants

Purposive sampling [19] was used to reach research participants who could provide insights into how their organization worked with peer-led sex education. The aim was to interview project leaders from NGOs who had been involved in the project’s inception, development, or implementation. Research participants were identified through contact information posted on project websites, or by contacting the NGOs. Potential research participants were contacted by telephone or email and informed about the study. Once contact was made, potential participants were sent an information letter including an invitation to participate. Those participants who were interested in the study and wanted to opt in were sent a second letter with detailed information about the study, the interview process and research consent forms. At the time of the interview, verbal information about the study was repeated, and they were again informed that participation is voluntary. Snowball sampling [19], whereby further eligible research participants were identified by initial participants, was used to recruit four of the participants. Out of the 15 participants that were initially identified, three declined participation and finally a total of twelve participants were interviewed for the study. These included seven women, four men and one non-binary person, whose age range was 26–45. All participants had post-secondary education in fields such as teaching, theater, community college pedagogy, social work, public health, and psychology. None of the project leaders reported having a cognitive disability which required an adapted work environment.

### Data collection

Individual in-depth interviews took place between July 2021 and August 2023. The interviews were held in person in university offices or project leaders work places. Two interviews were held online due to the COVID-19 pandemic restrictions. A thematic interview guide was used to probe predefined areas relevant to the research question. These thematic areas included SRHR, peer education, relationship to peer educators, and successes or obstacles to the project leaders work. Interviews lasted between 62–131 min and were voice recorded using a USB recorder. The interviews were performed, transcribed verbatim, and analyzed in Swedish. The interview opened by asking the participant to talk about their role in the project and to describe the project.

### Data analysis

The data was analyzed following Corbin and Strauss’ 2015 development of Grounded Theory, using a structured and systematic coding process [18]. To facilitate the initial open coding Nvivo software (1.7.1) was used to code the transcripts. Codes were constantly compared [18] within and across interviews, allowing for the identification of patterns and relationships, and then initially grouped under conceptual headings. The process of concept development and were recorded in memos as the concepts and categories evolved with new data [18]. Axial coding was used to explore the properties and dimensions of the concepts and resulting categories were constructed [18]. Finally, the overarching category was developed, and the categories were integrated into a conceptual model [18].

To achieve theoretical saturation, the authors defined categories based on their properties, dimensions, and variations, as guided by Corbin & Straus [18]. Documentation in terms of project reports and funding applications were then used to fill in the gaps of the final constructed coneptual model, as recommended by Corbin & Strauss [18]. Results were discussed among the authors. To further validate the model, a secondary analysis was performed by re-comparing the categories to the raw data. Furthermore, the researchers tested the synthesized data in the form of the emerging conceptual model with some of the participants in a group setting. Four of the participants who wished to participate discussed how the emerging model applied to their experience, as well as gave feedback to the research group about the terminology used within the categories. Following the session the research team reconvened, and the categories were reanalyzed and the wording adjusted.

### Ethical considerations

The study was approved by the Swedish Ethical Review Authority, reference number 2020–04440. No risk was anticipated for the participants; however, for anonymity of individuals and NGOs, all names and locations were removed from the transcribed data. Before the interviews began, research participants were informed about the study purpose and how the data would be handled. All research participants were informed that participation was voluntary and that they could stop the interview at any time without question. The interview files were stored on a password protected USB in a locked safe at the division of Social Medicine and Global Health, Lund University.

### Positionality statement

Our perspectives as researchers shape this study. The first author (BN) has a decade of experience in peer-led sex education for individuals with intellectual disabilities, bringing practical insight into the study context. ME and AA, both Professors in Global Health, contribute expertise in public health evaluation, SRHR, sexual coercion, and peer education. CL, Professor of Health and Society, specializes in sexuality and intellectual disability from a gender and social work perspective. MS, an Associate Professor, brings methodological expertise in youth and intervention research. We acknowledge that as educated researchers, our perspectives are shaped by privilege and academic frameworks that differ from the lived experiences of those who are the ultimate focal group of our research. While we bring expertise in qualitative research (including Grounded Theory), public health, peer-led education, SRHR and disability research we recognize the need for reflexivity to ensure our interpretations are centered in participant voices rather than dominant perspectives. We are committed to amplifying agency, addressing structural inequalities, and engaging with diverse perspectives to conduct ethical and meaningful research. We acknowledge our positions and remain reflexive in our approach to amplify participant voices.

## Results

**Fig. 1:**
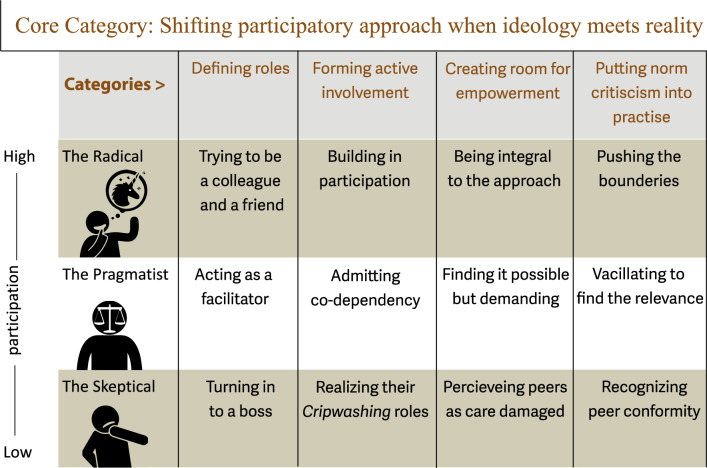
Conceptual model: approaches to peer-led sex education for and by persons with intellectual disability/autism

The analysis resulted in one core category ‘Shifting participatory approach when ideology meets reality’, which synthesizes the approaches project leaders take when working with peer-led sex education for and by persons with ID/ASD. Figure 1 represents a conceptual model of the relationship between three different approaches to peer- led sexual education, i.e. the Radical, the Pragmatic, and the Skeptical approach and four main categories identifying central components of these approaches 1) Defining roles 2) Forming active involvement 3) Creating room for empowerment, and 4) Putting norm criticism into practice. Sub-categories then indicate the properties of these categories depicting different strategies/insights used in the projects depending on the level of participation.

It should be noted that the participatory approaches are developed as theoretical constructs or “ideal types”, as described by Weber [20]. They are not static and project leaders move between different types of participatory approaches. Based on our data, project leaders often start off with a Radical approach, based on a strong ideological conviction about types and forms of participation, but when met with the reality of working with peer-led sex education for persons with ID/ASD, they at times shifted towards a more Skeptical approach, where the possibility of full participation by the peer-educators was questioned.

In the more detailed presentation, the results are presented under the headings of the main categories and with sub-headings indicating their properties. Quotes are provided to support how the interpretation is grounded in the data. In the citations, participants’ names have been replaced with numerical identifiers, 1–12.

### Defining roles

The first category in the model concerns defining the roles of the peer educator and project leader which are typically set by the NGO’s project leaders. Within a project, specific titles such as educator, expert, volunteer, and actor are used for the peer educators. These titles and role descriptions are related to the underlying ideologies the project leaders have.

#### Trying to be colleague and friend

In the Radical approach, project leaders aimed to be both colleagues and friends, striving to develop close relations to the peer educators: “…we became buddies, and we could kind of tune in on each other in a way*” (PL 8).* The project leaders had both collegial relations and friendships with the peer educators.

*...it was really not staff and participants like that, we were all colleagues - with the same rights or at the same level and that it was also very- it was very nice to have an equal relationship. (PL 2)*

Being friends and colleagues with the peer educators allowed project leaders to balance power dynamics and foster equality. Not only did the leaders take on roles where they developed collegial relationships to the peer educators, but they also viewed the peer educators as radical change catalysts. They described the peer educators as role models for other persons with ID/ASD, social geniuses, and experts on their own life. This view of the relationships can be seen as a shift in norms for social relations. Together these actors were redefining the relationships between persons with ID/ASD and those without a diagnosis.

#### Acting as a facilitator

In the Pragmatist approach, project leaders described their bond as a *semi relation* implying a mix of a personal and professional connection. “It's some kind of long-term friend/colleague relationship…we are in a sort of semi-relationship” (PL 9). They have developed a special relationship, but at the same time perceived themselves as personnel with a responsibility for the peer educators.

The facilitator’s role is to guarantee that practicalities are in place for a successful program. One participant observed that the facilitator’s involvement is limited, and that “their purpose is to simply uphold some type of structure” (PL 11). The facilitators do not have a leadership role nor are they participants; they are something in-between. In the Pragmatic approach, leaders chose peer educators for their leadership skills and professionalism during program implementation.

#### Turning into a boss

The Skeptical approach was characterized by project leaders who found the situations they were in required them to take on the role of a boss. Here the project leaders described an unequal power balance between the peer educator and themselves and explained how important it was to create a clear boundary when it came to roles in the program.

*…the fact that there can be a power inequality, [means] you need to be aware and create a safe space…So in general, boundaries should be maintained from a professional standpoint, which I also think, has to do with accessibility because it is about being clear. (PL 11)*

Within the Skeptical approach project leaders emphasized the importance of maintaining professional boundaries. However, this was something which they expected from themselves yet could not expect from the peer educators.

The Skeptical approach also meant questioning whether a peer really could be a representative for persons with a disability. The project leaders explained that this was a form of ableism, meaning that one’s ability is the determining factor to whether one can be a peer. According to the project leaders, peer educators who are high functioning, that can communicate clearly and maintain work, become peers, while those with who require more support are not fit to represent the group as a peer educator.

*It is also complex … not all [people who want to be peer educators] are suitable to go out and talk about sex…this has a lot to do with systemic ableism [Swedish: funktionsmaktsordningen]. It requires a certain level of [intellectual ability], for example, [one needs to have] impulse control to be able to work externally under [our NGO’s] name because we also have [the NGO’s] values that we need to stand for. … it is very complex who should [be selected as a peer educator]- because there are perks to work with us. (PL 6)*

Apart from ranking peer educators within a power system and prioritizing those with greater intellectual ability the quote also illustrates the power project leaders have to provide peer educators with access to employment and other benefits that come with working for an NGO.

### Forming active involvement

The second category illustrates that involvement of persons with ID/ASD was a central aspect of the projects. This involvement ranged from the inclusion of persons with ID/ASD in all stages of the project, to cripwashing, where persons with ID/ASD were seen as an alibi or a performative form of participation.

#### Building in participation

The Radical approach ensured that participation infiltrated all aspects of the project. Specifically, project leaders worked for and together with persons with ID/ASD. There was a conscious effort to shift power dynamics from those without ID/ASD to persons with ID/ASD and in this way increase participation. The project leaders explained that this is a radical way to approach SRHR work.

*... it permeated the whole project, you could say, in one way or another… to work with and for and together with the target group ...But it still felt so radical…that it was not "now we have done this for you," but "now you will also be a part of this" ...It felt a bit, revolutionary, sounds strange to say, but in a way I think it was, which it shouldn't be, but it also says quite a lot about the present times...So I think it's cool to have been part of that journey. (PL 8)*

A radical work environment guarantees equal employment opportunities for everyone*,* encompassing consistent pay, demands, and responsibilities. This was achieved through working with personalized tasks, based on one’s skill set. Peer educators had influence with regard to their roles, tasks, and modes of work. “In other words, all decisions were everyone's decisions…we didn't do anything without it being rooted in everyone*”* (PL 1). The Radical approach ensured that participation was a key value and signature throughout the project.

#### Admitting co-dependency

The Pragmatist approach acknowledged that participation created a co-dependency between the peer educators and the project leaders. Peer educators needed the project leaders to handle the bureaucracy involved in running projects, and the project leaders needed persons with ID/ASD to participate and form the very ideas the projects work from and with. Furthermore, many of the funders required focal group participation as a condition for funding.

However, the funders were also seen as giving conflicting messages. The project leaders pointed out that on the one hand they wanted persons with disability to be active in the projects, yet on the other hand persons with disabilities did not have the capacity to handle their administrative systems. Thus, it was not possible for persons with ID to run the projects on their own, hence the co-dependency.

*…[funders] point out how important it is with participation from the target group, but at the same time, nothing in their application is designed so that the target group themselves have opportunities to apply for it [funding]… sometimes feels like you can end up in a pretend participation...that really doesn't feel good. (PL 4)*

Some projects had variations of participation whereby persons with ID/ASD were volunteers or led peer circles. The project leaders acknowledged the benefits of having high degrees of participation but also admitted that this created administrative difficulties for them as project leaders. A solution to resolve the bureaucratic difficulties of paying wages to persons with ID/ASD was to change the roles of peer educators by reducing their level of participation in the projects.

*We have gone from having reference groups with a target group - where the target group really only had an opportunity to say what they might possibly want, to today we are much more of and for [the target group]. It is perhaps a development that many organizations have made and there are also demands from many funders, that there is a higher level of participation. That it's not just about having some teenage alibi in some panel debate, but that it's more about some kind of participation in the foundation of a project. (PL 10)*

The quote also illustrates the shift from using focal groups as performative participation in the form of reference groups or members of a panel debate, to ‘higher levels’ of participation, to where the focal group is the ‘foundation’ of a project. The Pragmatic approach championed robust participation, but also yielded to the challenges of Swedish bureaucracy, sometimes compromising participation for smoother project operations.

### Realizing their ‘*Cripwashing’* role

In the Skeptical approach persons with ID/ASD became more of surrogates for participation and figure heads for inclusion. Project leaders criticized the work environment and role which the peer educators with ID/ASD had in the projects.

*She was not safe in this process and she felt exploited...she was an alibi, that we prided ourselves on having a person from the target audience, but then she didn't think her opinions were worth anything. (PL 5)*

The project leaders likened this false participation to the way companies are criticized for false advertising referred to as green or pink washing. “Making one's voice heard, I think, is important for participation, so that it does not become…a pink washing or green washing” (PL 2*).* Furthermore, the leaders explained that it is complicated to realize genuine participation: “getting voices heard can be challenging, of course, but it feels like when you do manage it, you’re just putting on a display. It's kind of like that—a bit like Cripwashing [Swedish Funkwashing]” *(PL 8).* The participants described a fake or “pretend” participation of persons with ID/ASD. Persons with ID/ASD were included in the projects, not because they enhance the work but rather to appease funders requirements for participation of the focal group in the projects.

### Creating room for empowerment

The third category captured the levels of aspirations for a workplace where everyone could develop equally and succeed. The view empowerment ranged from equal work environments regardless of one’s ability, to focusing on achieving project goals, to working with coddled peer educators without common ground.

#### Being integral to the approach

In the Radical approach, project leaders described a work culture where everything and everyone worked on the same premise, an equal workplace. Project leaders felt that they were doing something important. They explained that the work was a mutual effort and described the personal development, both for themselves and the peer educators. They were proud not only of how they developed professionally as a project leader but also of how the peer educators developed and improved their sense of self. One project leader called this process the *empowerment effect,* meaning that the peer educators identified with their role as a peer educator, which gave them strength and possibility to create positive effects in other aspects of their lives.

*If people with disabilities…are group leaders or lead a group conversation, that conversation often leads much further and becomes much more meaningful and has a very strong 'empowerment effect.' (PL 7)*

When it came to mutual development, it was important to find areas where the peer educators and project leaders could meet each other on the same plane. One project leader explained how they let themselves be vulnerable with the peer educators, by showing what is also challenging for them.

#### Finding it possible but demanding—the show must go on

In the Pragmatic approach project leaders balance creating a work environment where peer educators worked on their own terms while at the same time achieving the project’s goals. The project leaders were driven by their own work ethic and the methods that they were implementing in the projects.

*...We're schooled that the show must go on…You've always been taught… that you don't just stay at home if you have a cold something like that…I think that all of us who come from that [schooling] have a rather strong sense of duty… that you implement what you have said…When you work with an external goal…it can be that you bend over backwards just to get the pieces to fit. (PL 4)*

This approach reflected the balancing act between creating a working environment where peer educators could flourish while at the same time maintaining the project leaders work ethic and desire to accomplish what the project set out to achieve. Sometimes this balance was difficult to find, and the project leaders found themselves in conflict with what they saw were the peer educators’ needs.

#### Perceiving peers as ‘care damaged’

In the Skeptical approach to peer education, project leaders’ demands on the peer educator could lead to conflict. One project leader described persons with ID/ASD as “care damaged, there are always people taking [care]- it’s always about one’s own needs istead of supporting each other” (PL 7). They portrayed a situation where persons with ID/ASD are raised in a system of institutions, special needs schools, and receive support through the LSS. This systematic support creates a society where the person with ID/ASD has little perspective beyond their own needs, which may lead to persons with ID/ASD becoming more *disabled* than what they are in reality. Instead, they are disabled by the system that is intended to help them.

### Putting norm criticism into practice

The fourth and last category encompasses the different approaches use of norm criticism, i.e. challenging societal standards and reflecting on ones impact on individuals and situations. Norm critical thinking characterized all three approaches’ communication with peer educators as well as the methods they used in the projects.

#### Pushing the boundaries

The radical approach incorporated norm criticism in all aspects of the project. The project leaders ensured that all sexualities, genders, ethnicities, and abilities were visible in the work environment as well as the materials. “It felt like not doing something new that wasn't norm-critical felt fucking wrong. So, it was just like this- it just couldn't be anything other than that” (PL 8). Part of being norm critical was to stretch the boundaries and societal norms of what sex for persons with ID/ASD was. Their aim was to create projects that had a positive view of sexuality with a strong focus on SRHR rights. They used theater, film, lectures, games, stories, interactive workshops, and pictures to lift the breadth of sexuality and present a lust filled meeting place, space, show, material, or experiences for individuals.

*Well, it was also just that, to create something that was between a biology book and a porn film. So, we made pictures that are so lovely that you think they are erotic without them being pornography. (PL 5)*

In this approach project leaders found ways to relate to the peer educators’ struggle of being outside functionality norms, by relating to their own breaking of gender and sexual identity norms.

*Our personal commitment to these issues, to also be able to partially identify as norm-breakers. If you are a norm-breaker in any way, I think it is easier to identify with other norm-breakers and unite in it,—in their struggle. (PL 5)*

The radical approach was characterized by passion to secure SRHR for persons with ID/ASD. However, this passion also involved a degree of critical reflection regarding the influence of societal norms on their methods and relationships between persons with ID/ASD and without.

#### Vacillating to find the relevance

In the pragmatist approach the leaders questioned whether their approach was motivated by an actual need. When describing the importance of using norm critical perspectives, a leader paused and then questioned why they were doing the project at all "after all, no one from the target group has directly asked us to do this” (PL 5). Moreover, leaders expressed uncertainty with regard to participants’ reception of their message. When explaining the methods used one research participant said that sometimes “I don't know how relevant that actually was to the people we worked for" (PL 3). On the one hand the project leaders believed in their methods, and on the other hand they struggled to acknowledge whether it was what persons with ID/ASD needed or wanted.

#### Recognizing peer conformity

In the Skeptical approach, leaders found it challenging to maintain a norm-critical view. They theorized that persons with ID/ASD, already deviating from functional norms, might hesitate to challenge sexuality norms. Project leaders described peer educators as adhering to sexuality and gender norms, likely as a way to counterbalance their departure from intellectual norms. “They [peer educators] also try to mask their disabilities, and some may exclusively do so, try to pass as normal as possible” (PL 5). The Skeptical approach suggested that persons with ID/ASD strive to integrate into society and often prefer to conceal their disability, aiming for a normative life.

## Discussion

The aim of this research was to explore the concept of peer education from the perspective of those managing such projects and to understand the ways in which peer-led sex education involving persons with ID/ASD is experienced, explained, reasoned about, and performed by project leaders employed by Swedish NGOs.

The findings are illustrated in the identified core category, *Shifting participatory approach when ideology meets reality*. Three approaches to peer-led sexual education were identified, i.e., the Radical, the Pragmatic, and the Skeptical approach characterized by different ways of *Defining roles*, *Forming active involvement*, *Creating room for empowerment*, and *Putting norm criticism into practice*.

The Radical approach operated on a flat power structure, with project leaders taking on roles akin to colleagues or friends. This ensured active participation throughout the projects. A key concept in this approach was “Empowerment”. Askheim [21] defines empowerment as both a process and an outcome; together this enables individuals to have greater control over their circumstances. Project leaders' views of empowerment aligns with Askehim’s definition, as they emphasize its potential to enhance SRHR for persons with ID/ASD.

Empowerment is highlighted as a crucial factor in peer education. An Australian study by Frawley and Bigsby (2014) on peer education for people with ID, supports this idea, as peer educators in their study reported feelings of empowerment. They demonstrated increased self-efficacy, power and control in their private relationships [22]. In a study by Garbutt [23] it was suggested that finding creative ways, not unlike those used in the radical approach, to ‘give voice’ to inform policy on sex and relationships resulted in persons with ID feeling more respected and empowered. On the other hand, Schnellert et al*.* [24] problematize the very foundations of empowerment and ‘giving voice’, claiming that these processes maintain the power inequality [24]. The data in the current study revealed that project leaders believed that their work empowered individuals to become active agents of change, both in their personal lives in general and in their roles as peer educators in particular.

In the Pragmatic approach project leaders adopted a facilitating role in the peer-led sexual education project. They acknowledged their codependence on individuals with disabilities to achieve the project's objectives. While they believed in the potential of empowerment, they also recognized that the project must progress. They questioned the methods applied within the context of persons with ID/ASD, and they sought a balance between the project's goals and the needs and capabilities of the peer educators. Within the Pragmatist approach leaders aimed to find workable solutions, considering both the aspirations of empowerment and the necessity to deliver a project efficiently. The project leaders acknowledged that the funders required applications, with goals and indicators which measure the value of a project in relation to the funding given to the project [25]. Furthermore, funders required ID/ASD participation, while those with ID/ASD relied on leaders for support and assistance in project management. The external pressure to achieve project goals and satisfy the funders’ needs puts external pressure on the project leader, which in practice shifts focus away from other, less tangible outcomes of the projects, such as self-confidence and self-development of the peer educators [12].

Swedish research on disability politics highlights how new public management in social welfare organizations mirrors profit-driven companies, emphasizing clear goals and success indicators [12, 26, 27]. The pragmatist approach seeks to address the dichotomy, on the one hand supporting the ideals as described by the radical typology and on the other hand being constrained and obligated to meet funder expectations. Mery Karlsson [12] would argue that in this approach the leaders have been through a co-optation process. At first, they were guided by strong principles, but to appease the financers they shifted the project goals from being ideologically motivated to become results based. The Pragmatist approach is, thus, characterized by a balancing act between a visionary ideology of the potentials of peer-led sex education and the reality of working with persons with ID/ASD. When this pragmatist approach moves too far away from the ideology of the Radical approach, the leader slides towards the more Skeptical approach.

In the Skeptical approach, project leaders acted more as bosses than facilitators, viewing the inclusion of individuals with disabilities in the education as "Cripwashing" (Swedish Funkwashing). The term refers to the phenomenon whereby persons with disabilities are used in media and public relations to evoke sympathy, yet any meaningful action is lacking [28]. At times the project leaders doubted the abilities of peer educators with ID/ASD, perceiving them as "care-damaged" and incapable of taking on the responsibilities required to manage the role as a peer educator. As a result, they believed that neither empowerment nor independence for certain individuals were possible. These perceptions of peer educators have the potential to undermine not only the relationships between peer educators, project leaders, and NGOs but also the overall foundation of the projects. Bigby and colleagues [29] found in their review that funding stakeholders in Australia and the UK doubted the suitability of persons with ID/ASD to be peer educators in targeted projects.

In the Skeptical approach, leaders tended to hold the belief that persons with ID/ASD are inherently different from others and do not wish to challenge norms. They may see individuals with disabilities as wanting to blend in with the mainstream society rather than embracing their unique identities. Project leaders within this approach claimed that peer educators did not wish to push boundaries, but they rather wished to conform.

This study did not use any specific predetermined theories for guiding the interviews, the initial coding, or in the development of the conceptual model. However, a theory that we have found useful to further increase our understanding of our conceptual model and the three peer education approaches is the Pedagogy of the Oppressed [30]. This theory questions the power dynamics inherent in classism and argues that the only way to overcome this system is if the oppressed persons, in Freire’s work the poor, through knowledge will revolt against the oppressors and free both themselves and the oppressors [30]. Freire [30] provides a framework for understanding the power relations between the project leaders and peer educators.

Askheim [21] argues that Swedish Social welfare users must challenge oppressive systems to achieve equal citizenship. Freire [30] emphasizes the need for liberating education that empowers individuals to critically analyze their social conditions and actively participate in transforming and eventually overthrowing oppressive systems. In the context of peer-led sexual education, the Radical approach’s commitment to empowerment aligns with Freire's pedagogical principles, as it seeks to create a collaborative and inclusive working and learning environment. As in the pedagogy of the oppressed the aim of the Radical approach is to boost agency and foster social change. In integrating empowerment project leaders working within this approach, as portrayed in “Creating room for empowerment”, believed they could challenge disabling conditions and create a more inclusive and equitable space for the expression of sexuality by persons with ID/ASD in society. By nurturing a sense of agency and self-advocacy in participants, this approach can foster a genuine co-creation of knowledge, which is also a key aspect in Freire’s pedagogy.

In summary, peer-led sex education programs prioritizing empowerment and social transformation can foster agency and confidence amongst participants. In turn participants can advocate for their sexual rights, challenge social barriers, and create a more inclusive and progressive SRHR and disability movement in Sweden. In the Radical approach leaders foster a high level of engagement and involvement among participants; they strive to create an inclusive environment for everyone involved in the peer-led sexual education project. As project leaders encounter complexities while working with peer educators, they at times transition from a Radical towards a more Pragmatic approach. While leaders in the Radical approach are driven by their commitment to empowerment and social transformation, the leaders in the Pragmatic approach focus on practical solutions and adapting strategies to ensure the project's progress and attainment of measurable outcomes. The Skeptical approach becomes a necessity for project leaders’ success and balance in the inherently demanding work environment.

The findings of this study have significant implications for the effectiveness of peer-led sex education programs. The use of different approaches provides a necessary balance by focusing on both empowerment and social change and practical aspects and feasibility. The combination of Radical, Pragmatic, and Skeptical approaches can lead to comprehensive and effective peer-led sex education initiatives for persons with ID/ASD. Nevertheless, the use of these seemingly contradictory approaches can lead to a dilemma and tension for the project leaders and peer educators.

### Methodological considerations

This study aimed to construct a conceptual model of approaches to peer-led sex education for and by persons with ID/ASD, based on how project leaders explain and reason about its application in their daily work. In qualitative research, credibility assesses whether the data and process of analysis address the intended focus of the research [31]. This study used direct quotes from participant interviews to enable readers to evaluate the analysis according to what was said by participants. A further measure of credibility was the use of member checks with research participants [32]. As explained in the methods, the researchers tested the synthesized data in the form of the emerging conceptual model, with some of the participants in a group setting. The participants, discussed how the emerging model applied to their experience, and gave feedback to the research group on the terminology used within the categories. The first author is employed in one of the NGOs included in the research, thus creating a potential source of bias. To minimize this, the research team held in-depth discussions on the research conception, process, and results. According to Malterud contesting each other’s interpretations and preconceived notions is a form of reflexive analysis which helps account for potential biases [33]. The fourth author performed interviews with the first author’s colleagues to increase proximity to the research participants. Furthermore, the dialogue within the research team enhances the dependability, as the research team can address potential design or phenomenon induced changes [31]. To enhance the transferability a distinct description of the study context was provided in the introduction. According to Drikso transferability is possible when sufficient contextual information is provided, and the applicability of the concept or theory is clearly defined [34].In this study the development of a conceptual model enables analytic generalization. In the model the data was separated from the individual participants temporal and spatial reality, thus increasing the transferability to similar settings and contexts to which this study took place in.

This project is part of a larger project which is also studying peer educators with ID/ASD and recipients of programs. Further research with peer educators themselves and recipients of peer education will aid in a holistic understanding of peer-led sex education for and by persons with ID/ASD.

## Conclusions

This study explores project leaders’ perspectives on peer-led sex education programs in Sweden aimed at increasing SRHR among adults with intellectual disabilities. It identifies three approaches: Radical, Pragmatic, and Skeptical, each reflecting different levels of participation in defining roles, forming active involvement, creating room for empowerment and, putting norm criticism into practice. While project leaders often start with a Radical approach, prioritizing full participation, practical constraints frequently lead to a Pragmatic shift, balancing idealism with structural limitations. In some cases, a Skeptical approach emerges, questioning the feasibility of full participation and highlighting concerns about performative inclusion.

For peer-led SRHR education to move beyond tokenism, giving persons with ID/ASD active roles in shaping and delivering SRHR education while adapting participation to individual abilities is necessary. Balancing empowerment with practicality requires structured training for project leaders, aligning participatory ideals with funding and organizational realities. Finally, peer education must transition from short-term projects to long-term projects, integrating standardized methodologies and training to ensure consistent, high-quality implementation. Addressing these areas will make peer-led sex education more inclusive, effective, and sustainable.

## Data Availability

The data generated and analysed during the current study are not publicly available due privacy of the research participants but are available in a redacted from the corresponding author on reasonable request.

## Abbreviations

CSE: Comprehensive sexuality education
ID/ASD: Intellectual disability and/or autism spectrum disorder
LSS: The act concerning Support and Service for Persons with Certain Functional Impairments
NGO: Non-Governmental Organization
SRHR: Sexual and Reproductive Health and Rights
